# Development and Preliminary Validation of a Stroke Physical Activity Questionnaire

**DOI:** 10.1155/2019/6764834

**Published:** 2019-07-25

**Authors:** Thunyakamon Phusuttatam, Jittima Saengsuwan, Pajeemas Kittipanya-ngam

**Affiliations:** ^1^Department of Rehabilitation Medicine, Faculty of Medicine, Khon Kaen University, Khon Kaen, Thailand; ^2^Northeastern Stroke Research Group, Khon Kaen University, Khon Kaen, Thailand

## Abstract

**Objective:**

The aim of the current study was to develop and then to determine preliminary validity of a physical activity questionnaire specifically designed for ambulatory, chronic stroke patients.

**Methods:**

This cross-sectional study recruited 92 chronic stroke patients. In Phase I, the SPAQ was developed and its content validity index (CVI) determined. In Phase II, we examined (1) the concurrent validity of SPAQ vs. the International Physical Activity Questionnaire-Short Form (IPAQ-SF); (2) the convergent validity of SPAQ vs. the Functional Ambulation Category (FAC), vs. the six-minute walk test (6-MWT), vs. the timed up and go test (TUGT), vs. the Motricity Index, vs. the National Institutes of Health Stroke Scale (NIHSS), and vs. the Modified Rankin Scale (MRS); and (3) the discriminant validity of the SPAQ vs. the Montreal Cognitive Assessment (MoCA). The concurrent validity and convergent and divergent validity were analysed using Spearman's rank correlation coefficient. The agreement between the SPAQ and IPAQ-SF was assessed using the Kappa statistic.

**Results:**

The SPAQ has 12 items in 3 main components which covers low (7 items), moderate (3 items), and vigorous (2 items) physical activity. The SPAQ had a CVI of 0.93. Spearman's correlation coefficient (r_s_) for SPAQ vs. IPAQ-SF was 0.53 (p < 0.001). The SPAQ yielded substantial agreement with the IPAQ-SF (Kappa = 0.65). For convergent validity, the SPAQ had a moderate correlation with the 6-MWT, MI, NIHSS, FAC, TUGT, and MRS (p < 0.05). For discriminant validity, the SPAQ was not correlated with the MoCA (r_s_ = 0.061, p = 0.68).

**Conclusion:**

The SPAQ can be used to practically assess physical activity in chronic stroke patients, and it has acceptable psychometric properties which are comparable to other standard physical activity questionnaires. Future work should determine the validity of the SPAQ using an objective device such as an accelerometer.

## 1. Introduction

Stroke is a leading cause of death and disability worldwide and continues to have a high incidence in low- to middle-income countries [1]. There is ample evidence that stroke patients have low levels of physical activity and are more inactive than their healthy peers [2–4]. The inactivity of stroke patients may be the result of factors related to stroke (i.e., fatigue, lack of cardiovascular fitness, or coordination problems) together with personal factors (i.e., motivation or being elderly) [5–7].

One aim of stroke rehabilitation is to enable the stroke survivor to develop and maintain an active lifestyle that meets the recommended physical activity and exercise guidelines, so as to improve physical function and prevent stroke recurrence and other cardiac events [8]. Physical activity in patients after stroke has been shown to have a positive effect on poststroke depression [9, 10], social participation [11], health related quality of life [12], and cognitive function [13, 14]. Physical activity may prevent recurrent stroke and decrease poststroke mortality by modifying stroke risk factors (e.g., by decreasing blood pressure, decreasing the total cholesterol/HDL ratio, decreasing BMI, and improving aerobic capacity [15]). Physical inactivity is a stroke risk factor [16].

Since the questionnaire is a practical and relatively inexpensive way of assessing physical activity, one might expect that several questionnaires would have been developed to assess physical activity among stroke patients. In fact, few questionnaires have been developed that specifically assess physical activity in this population. The questionnaires currently in use among stroke patients include the Physical Activity Scale for the Elderly (PASE) [17], the Physical Activity Scale for Individuals with Physical Disabilities (PASIPD) [18], the Human Activity Profile (HAP) developed for people with pulmonary problems, and the International Physical Activity Questionnaire Short Form (IPAQ-SF) for the general population [19].

The aim of the current study was to develop and then to determine preliminary validity of a physical activity questionnaire specifically designed for ambulatory, chronic stroke patients with no communication problems

## 2. Methods

This was a cross-sectional study conducted at the Rehabilitation Unit, Srinagarind Hospital, Thailand, between December 2017 and October 2018. The study was reviewed and approved by the Khon Kaen University Ethics Committee for Human Research (Ref. HE 601395). All participants signed informed consent prior to participation.

## 3. Subjects

The eligible population comprised chronic stroke patients who had had a haemorrhagic or ischaemic stroke 3 or more months previously. Patient inclusion criteria were (a) age ≥ 18 years, (b) ability to walk at least 10 m, and (c) ability to communicate. Exclusion criteria were (a) severe perceptual or communication problems and/or (b) severe concurrent cardiac or pulmonary disease.

Based on an in-depth interview, 15 stroke patients were recruited for development of the preliminary version of SPAQ1 (i.e., the Pre-SPAQ1). Thirty patients were then recruited to pilot the questionnaire so as to determine any deficits and to improve understanding (Pre-SPAQ2). The Pre-SPAQ2 was then evaluated for content validity by 5 experts and the final SPAQ was developed thereafter. Finally, 47 patients were recruited to determine the validity of the SPAQ. The sample size for the validation study was determined assuming a correlation coefficient of 0.4 between the SPAQ and the International Physical Activity Questionnaire Short Form (IPAQ-SF). This assumption was based upon the correlation coefficient between the IPAQ-SF and the Global Physical Activity Questionnaires (GPAQ) of 0.45-0.65 [20]. We hypothesised that in stroke patients the correlation might be lower than the general population. Alpha was set at 0.05, and *β* at 0.2. The sample size formula was sample size = 0.5*∗*ln[(1+r)(1-r)] [21], which yielded a total sample size of 47 patients.

## 4. Study Protocol

The study was divided into two main phases: Phase I was development of the stroke physical activity questionnaire (SPAQ), and Phase II determination of the validity of the SPAQ.

*Phase 1* (development) comprised 4 steps.

**Step 1 .**

Defining the concept of physical activity after stroke based on five aspects (viz. daily activities, household activities, outdoor activities, exercise, and hobbies).

**Step 2 .**

Conducting in-depth interviews with 15 post-stroke patients according to the five preceding concepts. The preliminary version of SPAQ (Pre-SPAQ1) was then developed based on the in-depth interview.

**Step 3 .**

Improving the Pre-SPAQ1 with respect to the preliminary version of the SPAQ2 (Pre-SPAQ2) by using the Pre-SPAQ1 to interview 30 patients. The data were then analysed with respect to the level and frequency of each activity.

**Step 4 .**

Rating the Pre-SPAQ2 for content validity by five experts. Expertise was accepted if the general rehabilitation doctor, cardiac rehabilitation doctor, neurologist, physical therapist, or home care nurses had at least 5 years' experience caring for stroke patients. The final stroke physical activity questionnaire (SPAQ) was then developed with input from experts.

*Phase 2* (Validation) determined concurrent validity of the SPAQ with the International Physical Activity Questionnaire-Short Form (IPAQ-SF). The convergent and discriminant validity were determined using data from the SPAQ as correlated with (a) the six-minute walk test (6-MWT), (b) the timed up and go test (TUG), (c) the Motricity Index, (d) the Functional Ambulation Category (FAC), (e) the National Institute of Health Stroke Scale (NIHSS), (f) the Modified Rankin Scale (MRS), and (g) the Montreal Cognitive Assessment (MoCA).

## 5. Statistical Analysis

SPSS version 23.0 was used for statistical analysis (IBM SPSS Statistics for Windows, Version 23.0. Armonk, NY: IBM Corp.). Normality was tested using the Kolmogorov-Smirnov test. Data that deviate from a normal distribution are reported as median and interquartile range (IQR). The association of reported participation in different activities with gait aids was analysed using the chi-squared test. Fisher's exact test was used when the number in each cell of the table was less than 5. The Mann-Whitney U test was used to compare average time in each activity in patients who were able to independently walk or used a gait aid. Spearman's rank correlation coefficient (r_s_) was used to study the relationship between time reported at moderate and high intensity and measured variables such as NIHSS or Motricity Index: r_s_ between 0.1 and 0.3 indicated a weak relationship; 0.3 to 0.5 a moderate relationship; between 0.5 and 1 a strong relationship; and 1 a perfect relationship [22].

Agreement between the SPAQ and the IPAQ-SF was determined using the Kappa statistic. We classified patients into 2 groups according to whether or not they met (or did not meet) the Center of Disease Control and Prevention (CDC) guidelines for individuals to have moderate to vigorous physical activity at least 150 min/week [23]. We determined the amount of exercise based on the duration of moderate and vigorous physical activity indicated in the questionnaire. A Kappa between 0 and 0.20 indicated slight agreement; 0.21 to 0.40 fair agreement; 0.41 to 0.60 moderate agreement; 0.61 to 0.80 substantial agreement; and 0.81 to 1.00 almost perfect agreement [24].

## 6. Results

### 6.1. Phase 1: Development of the Questionnaire

The preliminary in-depth interview of 15 poststroke patients comprised 21 questions. The SPAQ (Pre-SPAQ1) covered daily living (3 questions), household activities (5 questions), outdoor activities (4 questions), exercise (6 questions), and hobbies (3 questions).

The Pre-SPAQ1 was then piloted in 30 poststroke patients to assess the comprehensibility of the questionnaires. Some of the questions were not always completed by several of the patients, so we either adjusted or reduced some questions. Finally, there were 12 questions (preliminary version of SPAQ (Pre-SPAQ2)) and we arranged the questions based on intensity of exercise as light, moderate, and vigorous (i.e. 7, 3, and 2 questions, respectively).

The Pre-SPAQ2 was then assessed for content validity by five experts. The mean content validity index was 0.93 (range 0.8 to 1.0). We then made additional adjustments to the questionnaire based on the expert comments to improve the comprehensibility of the questions.

### 6.2. Phase 2: Validation of the Stroke Physical Activity Questionnaire

In Phase 2, we had 47 poststroke patients (age 57.5 ± 10.1 years; BMI 24.7 ± 4.3 kg/m^2^; mean ± standard deviation). Most were men (72.3%). The median poststroke time was 16.5 months. Thirty-nine patients (83 %) had had an ischaemic stroke and 30 (63.8 %) experienced left hemiparesis. Most patients used gait aids (55.3 %) and 42.6 % had not exercised prior to the stroke (Table 1).

According to the questionnaire results, the respective median time for light, moderate, and vigorous physical activity was 590, 0, and 0 min/week in patients with gait aids and 895, 70, and 0 min/week in patients who walked independently. Patients who walked independently were more likely to participate in light housework, light gardening, grocery shopping, and moderate intensity exercise. Patients with gait aids spent significantly less time in light housework, gardening work, grocery shopping, and moderate intensity exercise compared to those who walked independently. There was only a small proportion of patients who participated in high intensity exercise; thus there was no significant difference between patients who walked independently and those who used gait aids. Time in physical activity was 18.7 % of awake time in patients who walked independently and 13.8 % in patients with gait aids. The time without activity was approximately 12.7 and 13.5 h/day of awake time, and inactive time accounted for 81.3 % and 86.2 % of awake time in patients who walked independently and in patients who used gait aids, respectively (Tables 2 and 3).

## 7. Validity

### 7.1. Concurrent Validity and Agreement of SPAQ vs. IPAQ-SF

Concurrent validity tests whether the new questionnaire correlated well with previously established measurements.  Table 4 shows moderate correlation (r_s_ = 0.53, p < 0.001) between time spent in moderate and vigorous physical activity (MVPA) as obtained from the SPAQ and International Physical Activity Questionnaire-Short Form (IPAQ-SF). The SPAQ identified nine patients who met the physical activity recommendation per the CDC whereas the IPAQ-SF identified 13 patients who met the recommendations. The kappa value was 0.65 (p < 0.0001) (Table 5).

### 7.2. Convergent Validity

Convergent validity tests whether theoretical constructs are indeed related to the questionnaire endpoint.  Table 6 shows the correlation between the SPAQ and different measures of motor strength (Motricity Index), ambulatory level (FAC), balance (TUG), aerobic fitness (6-MWT), stroke severity level (NIHSS), and disability level (MRS). The SPAQ yielded a moderate correlation with the 6-MWT (r_s_ = 0.45, p = 0.001), Motricity Index (r_s_ = 0.43, p = 0.002), and the FAC (0.37, p = 0.01). The SPAQ was inversely correlated with the NIHSS (r_s_ = -0.48, p = 0.001), MRS (r_s_ = -0.38, p = 0.008), and TUG (r_s_ = -0.36, p = 0.013).

## 8. Discriminant Validity

Convergent validity tests whether constructs that are theoretically expected not to be related to the endpoint of the questionnaire are, in fact, not related.  Table 6 (lower panel) shows the correlation between the SPAQ and the MoCA. No significant correlation was found between SPAQ and MoCA.

## 9. Discussion

Assessment of physical activity is of great importance when assessing patient status, particularly as a guide to interventions and follow-up after rehabilitation. The aim of the current study was to develop and then to determine preliminary validity of a physical activity questionnaire specifically designed for ambulatory, chronic stroke patients with no communication problems. We developed a questionnaire after conducting in-depth interviews and running a pilot. The result was a 12-question SPAQ tool covering the intensity, duration, and frequency of physical activity. The data obtained from the SPAQ revealed the time spent at each intensity of physical activity per week. Since patients typically spend more time doing low-intensity physical activities, there are more questions related to low-intensity physical activity than moderate- and high-intensity activities.

According to the questionnaire, we found that most patients engaged in low-level physical activity. Only one-fifth (21.2 %) of the patients met the WHO global recommendations for physical activity [23]. Patients reported a median of 5 min of moderate physical activity per week. Inactive time accounted for 81.3 % to 86.2 % of total awake time with an average of 12.7 to 13.5 hours of inactive awake time. Our findings are consistent with previous reports of stroke patients using an accelerometer where inactivity accounted for 81.6 to 84.1 % of awake time [25], and the average inactive awake time was 13 hours per week [12, 26].

Concurrent validity indicated a strong relationship (r_s_ = 0.53) between the SPAQ and IPAQ-SF, as found between the IPAQ-SF vs. GPAQ among normal subjects (r = 0.45 to 0.65) [20]. Such a correlation coefficient is considered acceptable, considering the physical activity questionnaire should have a correlation greater than 0.50 when determining the relationship with other standard questionnaires [27]. We also found substantial agreement between the SPAQ vs. IPAQ-SF (kappa = 0.65) in terms of recommended physical activity per the WHO global recommendations (150 min/week of moderate to vigorous exercise). As for convergent validity, we found that SPAQ had a moderate correlation with proposed components (viz. FAC, Motricity Index, MRS, TUG-T, 6MWT, and NIHSS).

The correlation of SPAQ vs. 6-MWT in this study (r_s_= 0.45, p = 0.001, 47 chronic stroke patients) was comparable to that of PASE vs. 6-MWT (r_s_ = 0.47, p < 0.001, 49 chronic stroke patients) [28] and higher than PASIPD vs. 6-MWT (r = 0.31, p = 0.057, 40 chronic stroke patients) [12]. Based on the recommended acceptable correlation coefficient of more than 0.30 for physical activity questionnaires and physical functioning and health variables [27], the SPAQ demonstrated an acceptable correlation in every domain.

We used the MoCA to study the discriminant validity, which aimed to examine whether measurements that are not correlated by theory were also not correlated in the study. The MoCA—the cognitive level—is theoretically not related to physical activity; thus, as expected, there was no correlation between SPAQ and MoCA in our study.

Our study had several limitations. Although the questionnaires and fitness levels were considered adequate for the assessment of concurrent validity, standard measures (e.g. accelerometers) should be used to confirm the concurrent validity, and a reliability study should be done. The questionnaire was developed and validated for only ambulatory stroke patients with no communication problems, so further validation of the questionnaire will be required if it is to be used among patients with communication problems.

## 10. Conclusion

The SPAQ can be used to practically assess physical activity among chronic stroke patients and it has comparable psychometric properties to other standard physical activity questionnaires. Future work should determine the validity of the SPAQ using an objective device such as accelerometer.

## Figures and Tables

**Table 1:** Patient demographic data (n = 47).

Variable	Value
Sex, male/female, n (%)	34 (72.3)/13 (27.7)
Age (y)	57.5 (10.1)
Education level, n (%)	
(i) Primary school	9 (19.1)
(ii) Secondary school	16 (34.0)
(iii) College	4 (8.5)
(iv) University	15 (31.9)
BMI - Body mass index (kg/m^2^)	24.7 (4.3)
Cerebral infarction/Cerebral haemorrhage, n (%)	39 (83)/8 (17)
Side of hemiparesis, Left/Right, n (%)	30 (63.8)/17 (36.2)
Time since stroke (months), median (IQR)	16.5 (53)
Comorbidity, n (%)	
(i) Diabetes mellitus	15 (31.9)
(ii) Hypertension	29 (61.7)
(iii) Dyslipidemia	28 (59.5)
Premorbid Physical activity, n (%)	
(i) Inactive	20 (42.6)
(ii) Regularly (≥ 5 d/week) low level activity at least 10 min	3 (6.4)
(iii) Aerobic exercise 20-60 min/week	2 (4.3)
(iv) Aerobic exercise 1-3 h/week	4 (8.5)
(v) Aerobic exercise > 3 h/week	18 (38.3)
Barthel Index, median (IQR)	100 (10)
NIHSS	4.5 (3.6)
MoCA	21.8 (4.8)
Upper extremity motricity index, median (IQR)	56 (54)
Lower extremity motricity index	66.4 (25.1)
FAC, n (%)	
(i) 3	2 (4.3)
(ii) 4	17 (36.2)
(iii) 5	28 (59.6)
MRS, n (%)	
(i) 0	11 (23.4)
(ii) 1	4 (8.5)
(iii) 2	26 (55.3)
(iv) 3	6 (12. 8)
Gait aid, n (%)	
(i) No gait aid	21 (44.7)
(ii) Single cane	3 (6.4)
(iii) Tripod cane	22 (46.8)
(iv) Walker	1 (2.1)
Six-minute walk distance (m), median (IQR)	181.9 (260.0)
Timed up and go test (second), median (IQR)	15.1 (25.5)

Values are mean (SD) unless otherwise indicated.

*n* number; *SD* standard deviation; *IQR* interquartile range; *MoCA* Montreal Cognitive Assessment; *FAC* Functional Ambulation Category; *MRS* Modified Rankin Scale.

**Table 2:** Reported physical activity per the SPAQ.

*Variable*	Number of patients engaged in activity	Average time (min/week)	Average time (min/day)
*Low intensity*	47 (100.0)	1010.6 (949.5)	144.4 (135.6)
Basic activities of daily living	47 (100.0)	230.1 (127.8)	32.9 (18.3)
Light housework	23 (48.9)	113.7 (279.2)	16.2 (39.9)
Light gardening work	16 (34.0)	38.7 (71.8)	5.5 (10.3)
Grocery shopping	19 (40.4)	125.3 (377.8)	17.9 (54.0)
Flexibility exercise	42 (89.4)	225.0 (263.2)	32.1 (37.6)
Leisurely walking	43 (91.5)	175.0 (185.9)	25.0 (26.6)
Leisurely cycling	21 (44.7)	102.7 (287.9)	14.7 (41.1)
*Moderate intensity*	24 (51.1)	80.1 (167.7)	11.4 (24.0)
Walking up and down stairs	20 (42.6)	29.3 (52.6)	4.2 (7.5)
Brisk walking	12 (25.5)	80.2 (276.0)	11.5 (39.4)
Moderate intensity aerobic exercise i.e. cycling for exercise	13 (27.7)	50.5 (141.2)	7.2 (20.2)
*High intensity*	11 (24.4)	35.7 (85.0)	5.1 (12.1)
Strengthening exercise such as weight lifting	10 (21.3)	26.6 (65.1)	3.7 (9.3)
High intensity aerobic exercise i.e. running, aerobic dance	3 (6.4)	9.2 (38.3)	1.3 (5.5)

**Table 3:** Reported physical activity per the SPAQ classified by gait-aid usage.

*Variable*	Number of patients engaged in activity, n (%)				Average time (min/week), median (IQR)			
	Total (n = 47)	Walk with gait aids (n = 26)	Walk ID (n = 21)	p value	Total (n = 47)	Walk with gait aids (n = 26)	Walk ID (n = 21)	p value
*Low intensity*	47 (100.0 %)	26 (100.0 %)	21 (100.0 %)	1.00	730 (735)	590 (497)	895 (965)	0.03

Basic activities of daily living	47 (100.0 %)	26 (100.0 %)	21 (100.0 %)	1.00	210 (70)	210 (70)	210.0 (280.0)	0.24

Light housework	23 (48.9 %)	7 (26.9 %)	16 (76.2 %)	0.001	0 (120)	0 (15)	70.0 (200.0)	0.001

Light gardening work	16 (34.0 %)	5 (19.2 %)	11 (52.4 %)	0.017	0 (40)	0 (0)	30 (175)	0.012

Grocery shopping	19 (40.4 %)	4 (15.4 %)	15 (71.4 %)	< 0.001	0 (120)	0 (0)	120 (195)	0.001

Flexibility exercise	42 (89.4 %)	22 (84.6 %))	20 (95.2 %)	0.24	140 (150)	140 (143)	140 (268)	0.83

Leisurely walking	43 (91.5 %)	25 (96.2 %)	18 (85.7 %)	0.20	105 (140)	105 (140)	105 (155)	0.96

Leisurely cycling	21 (44.7 %)	10 (38.5 %)	11 (61.5 %)	0.34	0 (105)	2.8 (14.6)	20 (103)	0.46

*Moderate intensity*	24 (51.1 %)	8 (28.6 %)	16 (96.2 %)	< 0.001	5 (70)	0 (10)	70 (233)	< 0.001

Walking up and down stairs	20 (42.6 %)	6 (23.1 %)	14 (66.7 %)	< 0.001	0 (35)	0 (1)	35 (80)	0.002

Brisk walking	12 (25.5 %)	4 (15.4 %)	8 (38.1 %)	0.08	0 (15)	0 (0)	0 (128)	0.04

Moderate intensity aerobic exercise i.e. cycling for exercise	13 (27.7 %)	3 (11.5 %)	10 (47.6 %)	< 0.001	0 (35)	0 (0)	0 (140)	< 0.001

*High intensity*	11 (24.4 %)	5 (19.2 %)	6 (28.6 %)	0.45	0 (0)	0 (0)	0 (35)	NA

Strengthening exercise such as weight lifting	10 (21.3 %)	5 (19.2 %)	5 (23.8 %)	0.70	0 (0)	0 (0)	0 (18)	NA

High intensity aerobic exercise i.e. running, aerobic dance	3 (6.4 %)	1 (3.9 %)	2 (9.5 %)	0.43	0 (0)	0 (0)	0 (0)	NA

Values are median (IQR) unless otherwise indicated. *n* number; *IQR* interquartile range.

**Table 4:** Correlation of the SPAQ with IPAQ-SF.

Variable	Spearman's rho	p value
7 days IPAQ-SF (min/week)	0.53	< 0.001

**Table 5:** Agreement between recommended physical activity met identified by the SPAQ and IPAQ-SF.

SPAQ, n (%)			
IPAQ-SF, n (%)	Not met recommended physical activity for health	Met recommended physical activity for health	Total
Not met recommended physical activity for health	33 (70.1)	1 (2.1)	34 (72.3 %)
Met recommended physical activity for health	5 (10.6)	8 (17.0)	13 (27.7 %)
Total	38 (80.9)	9 (19.1)	47 (100) (p < 0.0001)

*∗∗*Recommended physical activity for health: 150 min of moderate intensity physical activity or 75 min of vigorous physical activity, kappa = 0.78.

**Table 6:** Correlation between SPAQ and other variables.

Variable	Spearman's rho	p-value
6-MWT (m)	0.45	0.001
Total motricity index	0.43	0.002
FAC	0.37	0.011
NIHSS	-0.48	0.001
MRS	-0.38	0.008
TUG (sec)	-0.36	0.013
MoCA	0.061	0.68

*6-MWT* six-minute walk test;* TUG* timed up and go test;* FAC* Functional Ambulation Category; *NIHSS *National Institute of Health Stroke Scale;* MRS* Modified Rankin Scale; *MoCA* Montreal Cognitive Assessment.

## Data Availability

The data used to support the findings of this study are included within the article.

## Supplementary Materials

Supplementary Materials Supplementary material 1: Stroke Physical Activity Questionnaire Thai version. Supplementary material 2: Stroke Physical Activity Questionnaire English version.
