# Supplementary material for: Development and Preliminary Validation of a Stroke Physical Activity Questionnaire
Source: Stroke Res Treat. 2019 Jul 25;2019:6764834. doi: 10.1155/2019/6764834 (PMC6683788; doi:10.1155/2019/6764834)
Supplement: Supplementary Materials — Supplementary material 1: Stroke Physical Activity Questionnaire Thai version. Supplementary material 2: Stroke Physical Activity Questionnaire English version. [file 6764834.f1.zip › Supplementary material 2 Stroke Physical Activity Questionnaire English version.pdf]

Supplementary materials

Stroke Physical Activity Questionnaire (SPAQ)

This questionnaire asks about the time you spent doing mild, moderate, and/or vigorous activity in the last 7 days. Physical activities requiring muscular movement in your daily activities include physical work, household chores, commuting (by walking/running/cycling), or exercise. Please estimate the time and frequency doing each activity.

**Type of physical activity**	**Frequency and duration**	**Average time per week (min)**
**1.** Low physical activities (activities that take minimal physical effort during which you could breathe normally or be able to sing.)		
1.1 Daily living activities (e.g., grooming, bathing, dressing)	Duration: .... hours........min/day	
1.2 Light housework (e.g., cleaning house, sweeping, dish washing, tidying, washing clothes)	☐ No<br>☐ Every day:<br>Duration: .... hours........min/day<br>☐ Other:<br>Frequency: ....days/week<br>Duration: .... hours........min/day	
1.3 Light gardening (i.e., trimming trees and watering)	☐ No<br>☐ Every day:<br>Duration: .... hours........min/day<br>☐ Other:<br>Frequency: ....days/week<br>Duration: .... hours........min/day	
1.4 Grocery shopping	☐ No<br>☐ Every day:<br>Duration: .... hours........min/day<br>☐ Other:<br>Frequency: ....days/week<br>Duration: .... hours........min/day	
1.5 Range of motion and flexibility exercise	☐ No<br>☐ Every day:<br>Duration: .... hours........min/day<br>☐ Other:<br>Frequency: ....days/week<br>Duration: .... hours........min/day	
1.6 Leisurely walking	☐ No<br>☐ Every day:<br>Duration: .... hours........min/day<br>☐ Other:<br>Frequency: ....days/week<br>Duration: .... hours........min/day	
1.7 Leisurely cycling	☐ No<br>☐ Every day:	

	Duration: .... hours........min/day<br>☐ Other:<br>Frequency: ....days/week<br>Duration: .... hours........min/day	
	Total time in low physical activities	
**2.** Moderate physical activities (activities that require moderate physical effort and you are breathing harder and faster than normal. You can talk in sentences but cannot sing.)		
2.1 Walking up and down stairs	☐ No<br>☐ Every day:<br>Duration: .... hours........min/day<br>☐ Other:<br>Frequency: ....days/week<br>Duration: .... hours........min/day	
2.2 Brisk walking	☐ No<br>☐ Every day:<br>Duration: .... hours........min/day<br>☐ Other:<br>Frequency: ....days/week<br>Duration: .... hours........min/day	
2.3 Moderate intensity aerobic exercise (i.e., cycling for exercise or others (describe)..........................	☐ No<br>☐ Every day:<br>Duration: .... hours........min/day<br>☐ Other:<br>Frequency: ....days/week<br>Duration: .... hours........min/day	
	Total time in moderate physical activities	
**3.** Vigorous physical activities (activities that take hard physical effort. You are breathing deeper and harder. You can speak 2 or 3 words but cannot speak in sentences.)		
3.1 Strengthening exercise (i.e., weight-lifting or resistance exercise)	☐ No<br>☐ Every day:<br>Duration: .... hours........min/day<br>☐ Other:<br>Frequency: ....days/week<br>Duration: .... hours........min/day	
3.2 High intensity exercise (i.e., running, aerobic dancing, or other high intensity exercise (describe)..................................	☐ No<br>☐ Every day:<br>Duration: .... hours........min/day<br>☐ Other:<br>Frequency: ....days/week<br>Duration: .... hours........min/day	
	Total time in vigorous physical activities	
