# Supplementary material for: Development and Preliminary Validation of a Stroke Physical Activity Questionnaire
Source: Stroke Res Treat. 2019 Jul 25;2019:6764834. doi: 10.1155/2019/6764834 (PMC6683788; doi:10.1155/2019/6764834)
Supplement: Supplementary Materials — Supplementary material 1: Stroke Physical Activity Questionnaire Thai version. Supplementary material 2: Stroke Physical Activity Questionnaire English version. [file 6764834.f1.zip › Supplementary material 2 Stroke Physical Activity Questionnaire Thai version.pdf]

แบบประเมินกิจกรรมทางกายในผู้ป่วยโรคหลอดเลือดสมอง (SPAQ)

<u>คำชี้แจง</u> : แบบประเมินนี้ เป็นการสอบถามเกี่ยวกับเวลาที่ใช้ในการเคลื่อนไหวร่างกายใน **7 วันที่ผ่านมา** เป็นการเคลื่อนไหวร่างกายในการทำกิจกรรมต่างๆทั้งหมดในแต่ละวัน เช่นขณะทำงาน ขณะอยู่บ้าน ขณะเดินทาง ขณะใช้เวลาว่าง และออกกำลังกาย หรือเล่นกีฬา เป็นต้น โดยจะขอให้ท่านประเมินความถี่และระยะเวลาในการทำกิจกรรมนั้น

รายละเอียดของกิจกรรม	ระยะเวลา	ระยะเวลาเฉลี่ยต่อสัปดาห์ (นาที)
**1. กิจกรรมระดับเบา** (กิจกรรมที่ใช้การออกกำลังกายน้อยทำให้ระดับการหายใจปกติระหว่างทำกิจกรรมนั้น สามารถพูดได้เป็นประโยคหรือร้องเพลงระหว่างทำกิจกรรมได้)		
1.1 การดูแลตนเองในการทำกิจวัตรประจำวัน ได้แก่ การล้างหน้า แปรงฟัน โกนหนวด การอาบน้ำ แต่งตัว เป็นต้น	วันละ .... ชั่วโมง........นาที	
1.2 การดูแลทำความสะอาดบ้าน การกวาดถูบ้าน การล้างจาน การจัดข้าวของในบ้าน การซักเสื้อผ้า	☐ ไม่ได้ทำ<br>☐ ทุกวัน วันละ ....ชั่วโมง........นาที<br>☐ อื่นๆ<br>ความถี่: ....วันต่อสัปดาห์<br>ระยะเวลา: วันละ........ ชั่วโมง........นาที	
1.3 การทำสวน เช่น การตัดเล็มต้นไม้ การรดน้ำต้นไม้	☐ ไม่ได้ทำ<br>☐ ทุกวัน วันละ ....ชั่วโมง........นาที<br>☐ อื่นๆ<br>ความถี่: ....วันต่อสัปดาห์<br>ระยะเวลา: วันละ........ ชั่วโมง........นาที	
1.4 การเดินซื้อของ จ่ายตลาด	☐ ไม่ได้ทำ<br>☐ ทุกวัน วันละ ....ชั่วโมง........นาที	

	☐ อื่นๆ<br>ความถี่: ....วันต่อสัปดาห์<br>ระยะเวลา: วันละ........ ชั่วโมง........นาที	
1.5 การออกกำลังกายเพื่อเพิ่มพิสัยข้อหรือความยืดหยุ่นของกล้ามเนื้อ	☐ ไม่ได้ทำ<br>☐ ทุกวัน วันละ ....ชั่วโมง........นาที<br>☐ อื่นๆ<br>ความถี่: ....วันต่อสัปดาห์<br>ระยะเวลา: วันละ........ ชั่วโมง........นาที	
1.6 การเดินเล่นและการเดินออกกำลังกายภายในบ้าน	☐ ไม่ได้ทำ<br>☐ ทุกวัน วันละ ....ชั่วโมง........นาที<br>☐ อื่นๆ<br>ความถี่: ....วันต่อสัปดาห์<br>ระยะเวลา: วันละ........ ชั่วโมง........นาที	
1.7 การปั่นจักรยานเล่นเพื่อความเพลิดเพลิน	☐ ไม่ได้ทำ<br>☐ ทุกวัน วันละ ....ชั่วโมง........นาที<br>☐ อื่นๆ<br>ความถี่: ....วันต่อสัปดาห์<br>ระยะเวลา: วันละ........ ชั่วโมง........นาที	
	ระยะเวลาโดยรวมของกิจกรรมระดับเบา	
**2. กิจกรรมระดับปานกลาง** (กิจกรรมที่ใช้กำลังกายปานกลาง ทำให้มีระดับการหายใจค่อนข้างเร็วระหว่างทำกิจกรรม สามารถพูดได้เป็นประโยคแต่ไม่สามารถร้องเพลงได้)		
2.1 การเดินขึ้นลงบันได	☐ ไม่ได้ทำ	

	☐ ทุกวัน วันละ ....ชั่วโมง........นาที<br>☐ อื่นๆ<br>ความถี่: ....วันต่อสัปดาห์<br>ระยะเวลา: วันละ........ ชั่วโมง........นาที	
2.2 การออกกำลังกายโดยการเดินเร็วๆ	☐ ไม่ได้ทำ<br>☐ ทุกวัน วันละ ....ชั่วโมง........นาที<br>☐ อื่นๆ<br>ความถี่: ....วันต่อสัปดาห์<br>ระยะเวลา: วันละ........ ชั่วโมง........นาที	
2.3 การปั่นจักรยานเพื่อการออกกำลังกาย และการรำกระบอง หรือการออกกำลังกายอย่างอื่นที่มีความหนักปานกลาง (ระบุ)..	☐ ไม่ได้ทำ<br>☐ ทุกวัน วันละ ....ชั่วโมง........นาที<br>☐ อื่นๆ<br>ความถี่: ....วันต่อสัปดาห์<br>ระยะเวลา: วันละ........ ชั่วโมง........นาที	
	ระยะเวลาโดยรวมของกิจกรรมระดับปานกลาง	
**3. กิจกรรมระดับหนัก** (กิจกรรมที่ใช้การออกกำลังมาก ทำให้ระดับการหายใจลึกและเร็ว สามารถพูดได้เพียงคำสั้นๆ 2-3 คำ ปนกับการหยุดพักหายใจแต่ไม่สามารถพูดเป็นประโยคได้)		
3.1 การออกกำลังกายเพื่อเพิ่มความแข็งแรงของกล้ามเนื้อ เช่นการยกน้ำหนัก, การถ่วงน้ำหนักที่แขนหรือขาแล้วออกแรงต้าน	☐ ไม่ได้ทำ<br>☐ ทุกวัน วันละ ....ชั่วโมง........นาที<br>☐ อื่นๆ<br>ความถี่: ....วันต่อสัปดาห์<br>ระยะเวลา: วันละ........ ชั่วโมง........นาที	

3.2 การออกกำลังกายแอโรบิคแบบหนัก เช่น การวิ่ง การเต้นแอโรบิค หรือการออกกำลังกายอย่างอื่นที่มีระดับหนัก (ระบุ).................................	☐ ไม่ได้ทำ<br>☐ ทุกวัน วันละ ....ชั่วโมง........นาที<br>☐ อื่นๆ<br>ความถี่: ....วันต่อสัปดาห์<br>ระยะเวลา: วันละ........ ชั่วโมง........นาที	
	ระยะเวลาโดยรวมของกิจกรรมระดับหนัก	
